# Oral antidiabetic treatment in type-2 diabetes in the elderly: balancing the need for glucose control and the risk of hypoglycemia

**DOI:** 10.1186/1475-2840-11-122

**Published:** 2012-10-06

**Authors:** Peter Bramlage, Anselm K Gitt, Christiane Binz, Michael Krekler, Evelin Deeg, Diethelm Tschöpe

**Affiliations:** 1 Institut für Pharmakologie und präventive Medizin, Menzelstrasse 21, 15831, Mahlow, Germany; 2 Institut für Herzinfarktforschung Ludwigshafen an der Universität Heidelberg, Ludwigshafen, Germany; 3 Herzzentrum Ludwigshafen, Medizinische Klinik B, Ludwigshafen, Germany; 4 Bristol Myers Squibb, Medical Department, Munich, Germany; 5 Stiftung “Der herzkranke Diabetiker” in der Deutschen Diabetes Stiftung, Bad Oeynhausen, Germany; 6 Herz- und Diabeteszentrum Nordrhein-Westfalen in Bad Oeynhausen, Universitätsklinik der Ruhr Universität, Bochum, Germany

## Abstract

**Background:**

We aimed at identifying variables predicting hypoglycemia in elderly type 2 diabetic patients and the relation to HbA1c values achieved.

**Design:**

Prospective, observational registry in 3810 patients in primary care. Comparison of patients in different age tertiles: with an age < 60 (young, n=1,253), age 60 to < 70 (middle aged, n=1,184) to those ≥ 70 years (elderly, n=1,373). Odds Ratios (OR) with 95% confidence intervals (CI) were determined from univariable and multivariable regression analyses.

**Results:**

Elderly patients had a later diabetes diagnosis, a longer diabetes duration, better glucose control and more frequent co-morbid disease conditions. Overall 10.7% of patients experienced any severity hypoglycemia within the last 12 months prior to inclusion. Higher rates of hypoglycemia were observed in the elderly than in the young after adjusting for differences in HbA1c, fasting and post-prandial blood glucose (OR 1.68; 95%CI 1.16-2.45). This was particularly true for hypoglycemic episodes without specific symptoms (OR 1.74; 95%CI 1.05-2.89). In a multivariate model stroke / transitory ischemic attack, the presence of heart failure, clinically relevant depression, sulfonylurea use and blood glucose self-measurement were associated with hypoglycemic events.

**Conclusion:**

Elderly patients are at an increased risk of hypoglycemia even at comparable glycemic control. Therefore identified variables associated with hypoglycemia in the elderly such as heart failure, clinically relevant depression, the use of sulfonylurea help to optimize the balance between glucose control and low levels of hypoglycemia. Asymptomatic hypoglycemia should not be disregarded as irrelevant but considered as a sign of possible hypoglycemia associated autonomic failure.

## Introduction

Providing adequate antidiabetic pharmacotherapy in the elderly is challenging due to age related co-morbid conditions and geriatric issues such as a loss of sensitivity towards hypoglycemia [1,2]. Further it appears, that a proper balance between the benefits of blood glucose lowering and hypoglycemia is more difficult to achieve than in younger patients. Low *glycosylated hemoglobin A1c* (HbA1c) targets result in an increased risk for hypoglycemia, and some antidiabetic drugs have been reported to confer additional risk [3,4].

Guidance on how to actually adjust glucose levels in elderly patients is provided by the *European Society of Cardiology* (ESC) [5], the *German Society for Diabetes* (DDG) [6] and the *American Association of Clinical Endocrinology* (AACE) [7]. These recommend HbA1c targets of < 6.5% in general while the *European Society for the Study of Diabetes* (EASD) [8] and the *American Diabetes Association* (ADA) recommend a less strict HbA1c target of < 7.0% [9]. While these targets also apply to healthy older adults with a life expectancy of more than 5 years [10], an HbA1c < 8.0% is deemed to be sufficient in elderly patients with multiple co-morbidities, functional disabilities and / or limited life expectancy. Formal evidence for these recommendations is however lacking and specific characteristics of elderly patients with an increased risk for hypoglycemia have not been described [11].

The present analysis, based on data of the DiaRegis *(Diabetes Treatment Patterns and Goal Achievement in primary diabetes care)* registry [12-15], aims at determining patient characteristics and clinical variables in the elderly that are associated with an increased risk of hypoglycemia, taking into account specific age related issues such as co-morbid disease and underlying medical treatment.

## Methods

DiaRegis is a prospective, observational, national, multicenter registry. It is conducted in accordance with the ethical principles that have their origin in the Declaration of Helsinki and adhere to *International Conference on Harmonization Good Clinical Practice* (ICH GCP), *Good Epidemiology Practices* (GEP), and applicable regulatory requirements. The protocol of this registry was approved by the ethics committee of the Landesärztekammer Thüringen in Jena, Germany on March 4^th^ 2009. Patients being enrolled into this registry provided written informed consent.

### Patients

Patients included: Between June 2009 and March 2010 a total of 3810 patients with type-2 diabetes aged ≥ 40 years on oral mono or dual oral combination antidiabetic therapy (no injectables such as insulin and glucagon-like peptide 1 [GLP-1] analogues) were included in a consecutive fashion on a center (physician office) basis. An additional requirement was that the treating physician considered an adjustment of antidiabetic pharmacotherapy to be necessary.

Patients not included: Patients not under regular supervision of the treating physician, patients with type-1 diabetes, pregnancy, diabetes secondary to malnutrition, infection or surgery, with maturity onset diabetes of the young, known cancer or limited life expectancy, acute emergencies, participation in a clinical trial and patients with further reasons that make it impossible or highly problematic for the patient to participate and to come to the follow-up visits were excluded.

For the present analysis the total cohort of 3,810 patients was divided into age tertiles of almost equal size aiming to provide sufficient statistical power to the analyses and to define age groups that are quantitatively relevant for clinical practice. The tertiles were labeled as follows: Patients with an age of at least 70 years at baseline (referred to as the elderly), patients younger than 70 but at least 60 years (middle aged) and an age group with patients below 60 years (young).

### Documentation

All variables were obtained by the treating physicians indicating the presence of absence of the disease but not objectively verified. This may be perceived as a limitation of the present registry but was not possible based on time and financial constraints. Patient variables were entered by physicians or their nurses via a secure website directly into an electronic database at the *Stiftung Institut für Herzinfarktforschung*, Ludwigshafen, Germany. At this stage they were automatically checked for plausibility and completeness. Data from the self-administered paper based patient questionnaire were transferred to the Clinical Research Organization *Winicker Norimed GmbH*, Nürnberg, Germany and entered into the electronic database.

### Definition of hypoglycemia

Hypoglycemia was obtained on an anamnestic, retrospective basis (within the last 12 months prior to the baseline visit). It was classified as mild, moderate or severe. Patients with mild hypoglycemia were defined as being with or without specific symptoms but manageable without help. These were usually detected by self-measurements of blood glucose (<2.22 mmol/l; 40 mg/dl in any case; 2.22-2.78 mmol/l or 50 mg/dl in case of symptoms) [12,14]. Patients with moderate hypoglycemia experienced symptoms of hypoglycemia and required assistance from a second person (e.g. a relative or friend), but no attention of a medical professional was necessary. Patients with severe hypoglycemia were seeking medical attention or were admitted to hospital because of hypoglycemia.

### Statistical analysis

**Figure 1:**
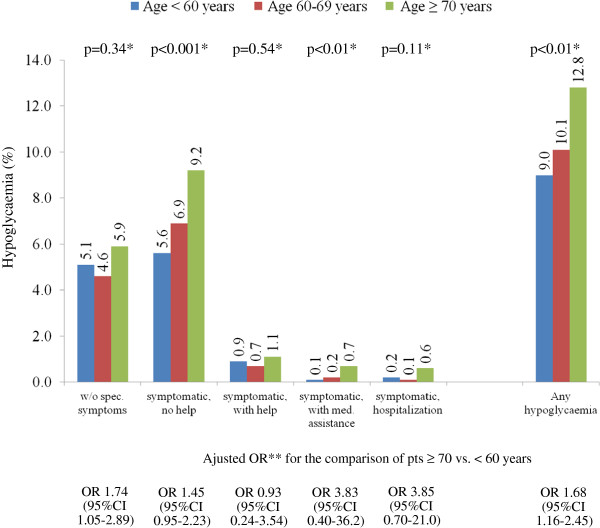
**Proportion of patients with at least one episode of anamnestic hypoglycemia per severity (12 months prior to baseline) in the elderly and the young.***Legend:* Odds ratios were adjusted for differences in baseline characteristics. * Cochran-Armitage or Jonchkheere-Terpstra test; **Adjusted for gender, concomitant diseases (CAD, PAD, heart failure and depression) and blood glucose levels (HbA1c, fasting and postprandial blood glucose).

**Figure 2:**
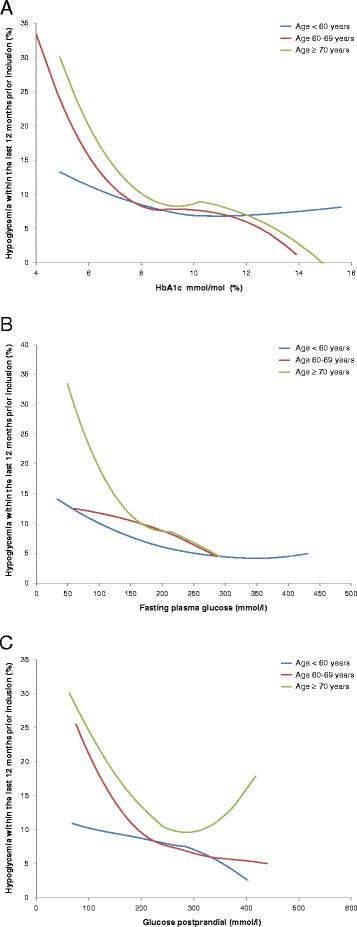
**HbA1c (A); Fasting plasma glucose (B); Postprandial plasma glucose (C) and anamnestic hypoglycemia (within 12 months prior to baseline).***Legend:* none.

The statistical analyses were performed using SAS version 9.2 (Cary, North Carolina, U.S.A.). All descriptive statistics are based on available cases. Patient characteristics (Table 1), concomitant disease (Table 1) and antidiabetic pharmacotherapy (Table 2) were analyzed with the Cochran-Armitage or Jonchkheere-Terpstra test. Adjusted Odds Ratios (ORs) for Figure 1 with 95% confidence intervals (CI) were derived from logistic regression analyses considering gender, concomitant diseases (coronary artery disease, peripheral arterial disease, heart failure and depression) and blood glucose levels (HbA1c, fasting and postprandial blood glucose). Multivariable logistic regression analysis was used to estimate adjusted ORs for Table 3 with 95%CI for the incidence of hypoglycemia within the 12 months prior to the baseline visit. Variables entered into the multivariable model were identified from univariable analysis and included age, female gender, HbA1c, fasting blood glucose, coronary artery disease, stroke / transitory ischemic attack (TIA), heart failure, depression, sulfonylurea (SU) use and blood glucose self-measurement. Figure 2 was produced using a robust method for smoothing by locally weighted regression (LOESS) with second order polynomials.

**Table 1:** Patient characteristics, laboratory values at baseline and co-morbid disease conditions

	**Age ≥ 70 years**	**Age < 70 to ≥ 60 years**	**Age < 60 years**	p-value*
	**(n=1,373)**	**(n=1,184)**	**(n=1,253)**	
	**Median (IQR) or %**	**Median (IQR) or %**	**Median (IQR) or %**	
Age (years)	74.0 (72.0-78.0)	65.0 (62.0-67.0)	54.0 (49.0-57.0)	<0.0001
Women (%)	51.1	45.9	42.5	<0.0001
Diabetes duration (years)	6.8 (3.9-10.6)	6.0 (3.3-9.5)	4.1 (1.9-7.3)	<0.0001
Physically active (any sport)	31.0	42.9	47.8	<0.0001
Body Mass Index (kg/m^2^)	29.0 (26.0-33.0)	31.0 (28.0-35.0)	32.0 (28.0-36.0)	<0.0001
Men (kg/m^2^)	29.0 (26.0-32.0)	30.0 (27.0-33.0)	31.0 (28.0-36.0)	<0.0001
Women (kg/m^2^)	30.0 (27.0-34.0)	32.0 (28.0-36.0)	33.0 (29.0-38.0)	<0.0001
Waist circumference (cm)	104 (96.0-113.0)	108 (98–117)	109 (99.0-120.0)	<0.0001
HbA1c (%)	7.3 (6.8-7.9)	7.5 (6.9-8.4)	7.6 (6.9-8.9)	<0.0001
Fasting plasma glucose (mmol/l)	7.7 (6.4-9.2)	7.8 (6.7-9.4)	8.1 (6.8-10.1)	<0.0001
Postprandial plasma glucose (mmol/l)	10.0 (8.6-11.8)	10.2 (8.4-12.2)	10.5 (8.7-13.0)	<0.0001
Co-morbidity				
Dyslipidemia	64.9	65.5	59.3	<0.01
Hypertension	90.5	86.2	76.0	<0.0001
Coronary heart disease	28.1	16.0	8.5	<0.0001
Myocardial infarction	28.8	37.0	50.0	<0.0001
Stable angina	33.6	26.2	20.0	<0.01
Unstable angina	3.2	3.8	5.1	0.40
PCI	35.2	44.1	52.5	<0.001
Bypass surgery	21.6	21.4	19.8	0.74
Prior stroke / TIA	6.8	4.3	2.5	<0.0001
Heart failure	18.6	7.5	2.6	<0.0001
Peripheral arterial disease	8.2	6.4	3.2	<0.0001
Amputation	1.3	0.6	0.8	0.16
Autonomous neuropathy	4.4	3.5	2.1	<0.01
Peripheral neuropathy	18.4	15.8	8.5	<0.0001
NPDR	5.2	3.5	2.4	<0.001
Proliferative retinopathy	1.0	0.4	0.1	<0.01

*Legend:* HbA1c, glycosylated hemoglobin A1c; PCI, percutaneous coronary intervention; TIA, transitory ischemic attack; NPDR, non-proliferative retinopathy; * Cochran-Armitage or Jonchkheere-Terpstra test.

**Table 2:** Antidiabetic pharmacotherapy at baseline before therapy adjustment per age group (combinations with a proportion < 1% were omitted)

	**Age ≥ 70 years**		**Age < 70 to ≥ 60 years**		**Age < 60 years**		p-value*
	**n=1,373, (%)**		**n=1,184, (%)**		**n=1,253, (%)**		
	**n**	**%**	**n**	**%**	**n**	**%**	
Oral monotherapy	973	70.9	777	65.6	867	69.2	0.32
Metformin	700	51.0	616	52.0	751	59.9	<0.0001
Sulfonylureas	205	14.9	101	8.5	81	6.5	<0.0001
Glucosidase inhibitors	27	2.0	22	1.9	15	1.2	0.13
Glinides	29	2.1	23	1.9	9	0.7	<0.01
Thiazolidinediones	5	0.4	11	0.9	6	0.5	0.66
DPP-4 inhibitors	7	0.5	4	0.3	5	0.4	0.65
Oral dual combination	400	29.1	407	34.4	386	30.8	0.32
Met + SU	251	18.3	232	19.6	183	14.6	<0.05
Met + Glukos	12	0.9	12	1.0	6	0.5	0.26
Met + Glin	27	2.0	39	3.3	37	3.0	0.11
Met + Glitaz	47	3.4	62	5.2	75	6.0	<0.01
Met + DPP-4 inhibitors	39	2.8	49	4.1	63	5.0	<0.01

*Legend.* DPP-4, Dipeptidyl-peptidase 4 inhibitors; OAD, oral antidiabetic drug; * Cochran-Armitage or Jonchkheere-Terpstra test; No GLP-1 analogues or insulins were prescribed because patients with these antidiabetic drugs at baseline were excluded by the study protocol [12].

**Table 3:** Variables associated with anamnestic hypoglycemia in the elderly 12 months prior to inclusion

		**Univariable analysis**	**Multivariable analysis (fixed model)**	**Multivariable analysis (stepwise model)**
		**OR (95%CI)**	**OR (95%CI)**	**OR (95%CI)**
Female gender	(vs. male)	0.98 (0.71-1.34)	0.92 (0.63-1.35)	--
Diabetes duration	per 10 years	**1.30 (1.02-1.65)**	1.29 (0.97-1.71)	--
Coronary artery disease yes	(vs. no)	**1.62 (1.16-2.26)**	1.11 (0.72-1.71)	--
Stroke / TIA yes	(vs. no)	**2.10 (1.26-3.51)**	**1.94 (1.04-3.59)**	**1.96 (1.06-3.62)**
Heart Failure yes	(vs. no)	**1.68 (1.16-2.44)**	**1.61 (1.02-2.53)**	**1.63 (1.07-2.49)**
Depression yes	(vs. no)	**3.88 (2.33-6.47)**	**4.24 (2.35-7.65)**	**4.20 (2.36-7.46)**
Sulfonylurea yes	(vs. no)	**1.90 (1.38-2.62)**	**1.71 (1.17-2.49)**	**1.82 (1.25-2.63)**
BG self-measurement yes	(vs. no)	**2.17 (1.35-3.50)**	**1.92 (1.18-3.11)**	**2.00 (1.24-3.23)**

*Legend:* * median values for the cohort of elderly people were taken as the cut-off, corresponds to 7.3% or 138 mg/dl respectively.

## Results

### Difference in patient characteristics between elderly and younger diabetic patients

There were 1,373 elderly patients ≥ 70 years, 1,184 patients between 60 and < 70 years and 1,253 patients below 60 years (young) (Table 1). Elderly patients were more frequently female, had a later diabetes diagnosis and had longer diabetes duration. They were less frequently physically active (any sports) and had a lower body mass index (BMI) and waist circumference. Glucose control as indicated by HbA1c, fasting and postprandial glucose levels was significantly better in the elderly.

Elderly patients had a distinct co-morbidity profile: Hypertension, prior stroke / TIA, peripheral arterial disease, heart failure, autonomous and peripheral neuropathy were substantially more frequent (Table 1). This also applied to coronary artery disease; it was remarkable however, that a higher proportion of younger patients with coronary artery disease (CAD) already had experienced myocardial infarction (50.0 vs. 28.8%; p<0.0001), while there were more patients with stable angina in the elderly (33.6 vs. 20.0%; p<0.01). This was confirmed by a significantly more frequent use of renin angiotensin system blocking agents, beta blockers, calcium channel blockers, diuretics, antiplatelet agents and lipid lowering agents in the elderly (all differences at least p<0.01).

Finally there was a noteworthy difference in the use of antidiabetic drugs at baseline such as lesser use of metformin but a more frequent used of sulfonylureas and glinides in monotherapy or a lesser use of Met/Glitazone and Met/dipeptidyl peptidase 4 inhibitor (DDP-4 inhibitor) and a more frequent use of Met/SU in those with a dual combination therapy based on metformin (Table 2).

### Hypoglycemia vs. blood glucose control

11.0% of all patients reported to have had at least 1 episode (or more) of hypoglycemia in the 12 months prior enrolment. This was reported more frequently in elderly than in younger patients (12.8 vs. 9.0%; p<0.01) (Figure 1). Further significant differences were seen for symptomatic episode without a need for help (9.2 vs. 5.6%) and symptomatic episodes with a need for medical assistance (0.7 vs. 0.1%). After adjusting for differences in baseline characteristics such as gender, blood glucose and concomitant disease overall rates remained elevated (OR 1.68; 95%CI 1.16-2.45; p=0.0057 for trend across age groups) as were hypoglycemic episodes without specific symptoms (OR 1.74; 95%CI 1.05-2.89; p=0.0378 for trend across age groups).

In Figure 2A-C we plotted values for blood glucose control (HbA1c, fasting and postprandial plasma glucose) against the frequency of anamnestic hypoglycemia in the three different age groups. The figure illustrates higher rates of anamnestic hypoglycemia in the elderly at different HbA1c, fasting and post-prandial blood glucose levels. It appears that for HbA1c values < 7.5% and fasting blood glucose levels < 6.1-6.7 mg/dl the occurrence of hypoglycemia in the elderly is increasing more dramatically than in younger patients < 60 years.

### Variables associated with hypoglycemia in the elderly

Noteworthy differences between elderly patients experiencing hypoglycemia and those without were a higher prevalence of CAD, stroke / TIA, heart failure, depression, sulfonylurea use and blood glucose self measurement. In a stepwise multivariate model, considering these differences in baseline characteristics stroke / TIA (OR 1.96; 95%CI 1.06-3.62), the presence of heart failure (OR 1.63; 95%CI 1.07-2.49), clinically relevant depression (OR 4.20; 95%CI 2.36-7.46) and sulfonylurea use (OR 1.82; 95%CI 1.25-2.63) remained predictors of hypoglycemia in the elderly (Table 3). In addition blood glucose (BG) self-measurement led to a higher awareness of (subclinical) hypoglycemia (OR 2.00; 95%CI 1.24-3.23).

## Discussion

In this analysis elderly patients ≥ 70 years were more likely to have suffered from any severity (but mostly asymptomatic) hypoglycemia within the last 12 months prior to inclusion than younger patients < 60 years at comparable glycemic control. This is important because it should result in increased awareness of physicians and patients towards this severe but sometimes even asymptomatic complication and in an adjustment of treatment to prevent further complications.

### Hypoglycemia in the elderly

It is well known that elderly patients are at an increased risk for hypoglycemia and often limits their proper management [16]. Risk factors for hypoglycemia are similar to those in the young but are highly prevalent in the elderly. These include multiple co-morbidities, polypharmacy (≥ 5 medications), chronic renal or hepatic impairment, poor nutrition, use of sulfonylurea or insulin, acute illness, hypoglycemic unawareness and diminished counter regulatory responses [17].

Although it has been reported that hypoglycemia is more frequent in the young [18], rates of symptomatic hypoglycemia appear to be reduced in the elderly. This has been attributed to repetitive hypoglycemia leading to blunted symptomatic and hormonal responses to subsequent episodes leading to impaired awareness of hypoglycemia, also called *hypoglycemia associated autonomic failure* (HAAF) [19]. These patients often experience glucose concentrations below 2.0 mmol/l without becoming symptomatic. Furthermore, a number of variables such as glycemic control, alcohol, exercise, and age affects and reduces symptomatic and hormonal responses to subsequent hypoglycemia [20-24]. Elderly patients also report different symptoms and responses to hypoglycemia with less autonomic and more prominent neuroglycopenic symptoms [25]. In this group, hypoglycemia can be misdiagnosed as dementia or neurological events [26].

### Pharmacotherapy and hypoglycemia

Polypharmacy is an important predictor of subsequent hypoglycemic events [17]. This is exemplified in our study with a more frequent use of cardiovascular medical treatment (beta blockers and diuretics in particular). Both drugs not only add to polypharmacy but are considered to impair glucose control by reducing hypoglycemia awareness and countermeasures or to simply increase blood glucose levels directly [27,28]. Antidiabetic treatment was also different in the elderly, who were more frequently treated with sulfonylureas and less frequently with metformin, thiazolidinediones and DPP-4 inhibitors, a treatment pattern which was even more pronounced in elderly who experienced hypoglycemia during the last 12 months. The occurrence of hypoglycemia as a result of antidiabetic treatment of the elderly casts a cloud over modern risk adopted medical therapies and foils its achievement. Not surprisingly the uni- and also multivariate analyses found an elevated risk of hypoglycemia in those being treated with sulfonylureas. Taken together polypharmacy and in particular therapies with a remarkable hypoglycemic potential, such as sulfonylureas, should be used with caution, especially in patients with co-morbid disease such as heart failure or CAD, who require medication that might increase this risk for hypoglycemia.

### Variables associated with hypoglycemia in the elderly

Beyond the use of sulfonylureas, stroke / TIA, heart failure and clinically relevant depression were predicting an increased risk for hypoglycemia in a multivariate model. Interestingly also patients who perform blood glucose self-measurement had an increased risk of (asymptomatic) hypoglycemia. This may be regarded as a self-fulfilling prophecy but is important not only because asymptomatic biochemical hypoglycemia may result in neurological impairment but because severe hypoglycemia may be masked as being asymptomatic in the elderly [19]. Indeed we found that the majority of hypoglycemic events in the elderly were either asymptomatic or symptomatic but without the need for help.

Although the association of hypoglycemia with depression has already been described [29,30], it is a finding with major public health implications. In a study of 99 adult patients with long-standing type-1 diabetes it was shown that poor sleep quality was independently associated with a positive *hospital anxiety and depression scale* (HADS), a possible explanation could be the occurrence of nocturnal hypoglycemia [29]. Undisputable and self-explaining is the fact that hypoglycemia is causing reductions in health related quality of life as a study in type-2 diabetes mellitus patients showed and depression is a major determinant of this. Further research is warranted to evaluate if these mechanisms are solely able to explain the findings or if other variables should be taken into account [30].

### Blood glucose targets in the elderly

It is important to understand that especially the elderly gain benefit from an individualized approach, instead of undifferentiated efforts to lower blood glucose. The ADA generally considers an HbA1c < 8.0% as being sufficiently tight in elderly patients with multiple co-morbidities, functional disabilities and / or limited life expectancy [9]. The DDG proposes a more individualized approach and considers strict HbA1c values to be not very useful [31]. However, to give an orientation of how elderly patients should be treated in terms of their blood glucose and HbA1c targets, the 2010 DDG practice guidelines propose a decision making process based on the actual health situation of the patients and his/her functional status. Patients with a good functional status (no reduction of their autonomy, good self-management and training skills), and a low level of co-morbidity (so called ‘go-go’ patients) should aim for an HbA1c between 6.5-7.0% without hypoglycemia. Patients with a reduced functional status (reduced autonomy, self-management and training skills), and multi-morbidity (so called ‘slow-go’ patients) should aim for an HbA1c of 7.0-8.0% without hypoglycemia. Only patients with significant functional reductions or limited life-expectancy (so called ‘no-go’ patients) should not aim for any certain HbA1c level, rather than to avoid symptoms of diabetes and hyper- or hypoglycemia. In this group of patients the focus is to preserve of a maximal quality of life [31-33].

It is important to highlight that the database for sufficient evidence based decisions and an optimal treatment of the elderly diabetic patients is weak. More efforts are required to set up a solid database of this steadily increasing group of patients. The necessity to include more of the elderly into clinical trials on the treatment of diabetes and to perform functional and cognitive assessments accordingly is a challenging requirement of geriatric societies with a high prevalence of diabetes in order to optimize medical therapy [31].

### Results in perspective

Just recently a new consensus statement on the treatment of type-2 diabetes in the elderly was developed by the International Association of Gerontology and Geriatrics (IAGG), the European Diabetes Working Party for Older People (EDWPOP), and the International Task Force of Experts in Diabetes [34]. They stated that hypoglycemia is highly prevalent and underrecognized in older people and that longer-acting sulfonylureas (or insulin) confer an increased risk. In those at high risk sulfonylureas should be avoided and DPP-4 inhibitors or, in the case of a BMI > 35 kg/m^2^, GLP-1 analogues should be considered. In addition they recommend not to lower blood glucose too aggressively in the elderly. These recommendations are consistent with our own findings and the potential clinical implications of our work.

### Limitations

Despite the considerable strength of the study in documenting real world patients, treatment patterns, co-morbidity and treatment related events a few limitations of the present analysis deserve mentioning. 1) The present analysis only considered oral antidiabetic drugs for the evaluation of hypoglycemia in the elderly. Therefore it is consistent with prior data that we identified sulfonylurea but not insulin as being associated with events. 2) Hypoglycemic events were recorded on an anamnestic basis where physicians and patients were required to recall events within the last 12 months. The bias however appears to be reasonably confined because preliminary data for the first year of follow-up resulted in similar hypoglycemia rates. 3) While we also considered to look at the very elderly (80+) we chose a definition of 70+ as being elderly. This was because of the quantitative importance of this patient group which makes up almost one third of patients in clinical practice. 4) Clinical diagnoses on co-morbid disease conditions were not validated but relied on the physicians’ assessment instead. This is common practice in this type of registries and cannot be alleviated because of financial constraints and the acquisition of data in real world clinical practice and its well known constraints of time.

## Conclusions

Hypoglycemia is a serious clinical condition which impacts clinical outcome, even more so in the elderly with frequent concomitant diseases. Therefore identified variables associated with hypoglycemia in the elderly such as heart failure, clinically relevant depression, the use of sulfonylurea help to optimize the balance between glucose control and low levels of hypoglycemia. Asymptomatic hypoglycemia should not be disregarded as irrelevant but considered as a sign of possible hypoglycemia associated autonomic failure.

## Abbreviations

AACE: American Association of Clinical Endocrinology; ADA: American Diabetes Association; BG: Blood glucose; BMI: Body mass index; CAD: Coronary artery disease; CI: Confidence intervals; DDG: German Society for Diabetes; DiaRegis: Diabetes Treatment Patterns and Goal Achievement in primary diabetes care; EASD: European Society for the Study of Diabetes; DPP-4: Dipeptidyl peptidase 4; EDWPOP: European Diabetes Working Party for Older People; ESC: European Society of Cardiology; GEP: Good Epidemiology Practices; GLP-1: Glucagon-like peptide 1; HAAF: Hypoglycemia associated autonomic failure; HADS: Hospital anxiety and depression scale; HbA1c: Glycosylated hemoglobin A1c; IAGG: Association of Gerontology and Geriatrics; ICH GCP: International Conference on Harmonization Good Clinical Practice; IQR: Interquartile Range; NPDR: Non-proliferative retinopathy; OR: Odds Ratios; PAD: Peripheral artery disease; PCI: Percutaneous coronary intervention; SU: Sulfonylurea; TIA: Transitory ischemic attack.

## Competing interests

AKG, PB, ED and DT have received research support and honoraria for lectures from Bristol-Myers Squibb and AstraZeneca, the sponsors of the present registry. CB and MK are employees of the sponsors.

## Authors’ contributions

AKG, PB, DT, CB, and MK have been deeply involved in the conception and design of the study. ED is responsible for the analysis of data. PB has drafted the manuscript and all other authors have been revising the article for important intellectual content. All authors read and approved the final manuscript.
